# Lab-on-a-Bird: Biophysical Monitoring of Flying Birds

**DOI:** 10.1371/journal.pone.0123947

**Published:** 2015-04-16

**Authors:** Abdurrahman Gumus, Seoho Lee, Syed S. Ahsan, Kolbeinn Karlsson, Richard Gabrielson, Christopher G. Guglielmo, David W. Winkler, David Erickson

**Affiliations:** 1 School of Electrical and Computer Engineering, Cornell University, Ithaca, NY, United States of America; 2 Sibley School of Mechanical and Aerospace Engineering, Cornell University, Ithaca, NY, United States of America; 3 Applied and Engineering Physics, Cornell University, Ithaca, NY, United States of America; 4 Department of Ecology and Evolutionary Biology, Cornell University, Ithaca, NY, United States of America; 5 Department of Biology, Advanced Facility for Avian Research, University of Western Ontario, London, Ontario, Canada; Coastal Carolina University, UNITED STATES

## Abstract

The metabolism of birds is finely tuned to their activities and environments, and thus research on avian systems can play an important role in understanding organismal responses to environmental changes. At present, however, the physiological monitoring of bird metabolism is limited by the inability to take real-time measurements of key metabolites during flight. In this study, we present an implantable biosensor system that can be used for continuous monitoring of uric acid levels of birds during various activities including flight. The system consists of a needle-type enzymatic biosensor for the amperometric detection of uric acid in interstitial fluids. A lightweight two-electrode potentiostat system drives the biosensor, reads the corresponding output current and wirelessly transfers the data or records to flash memory. We show how the device can be used to monitor, in real time, the effects of short-term flight and rest cycles on the uric acid levels of pigeons. In addition, we demonstrate that our device has the ability to measure uric acid level increase in homing pigeons while they fly freely. Successful application of the sensor in migratory birds could open up a new way of studying birds in flight which would lead to a better understanding of the ecology and biology of avian movements.

## Introduction

Research on birds has served as an effective tool for ecological and evolutionary studies, as the metabolism of birds are finely tuned to their environments [1]. Development of new generations of radio-tags and data-loggers give the ability to track and study migratory birds by monitoring bird’s dynamic locations [2–4]. Some transmitters can also give information from sensors that provide information about the bird’s physiology such as heart rate and body temperature [5–7]. Continuous biophysical monitoring of birds, however, has been prevented by the absence of a sensor system that can make in vivo measurements on birds in flight. The current state-of-the-art for biophysical monitoring of birds is to take blood samples from the captured birds for their analysis in remote laboratories at later times [8–12]. This method only yields sporadic measurements of the birds’ physiological state and is likely to be affected by the interrupted and disruptive sample-collection procedures.

Enzymatic needle-type in vivo biosensors have the potential for a more comprehensive and accurate analysis of physiological state by allowing continuous measurements in real time. Previously, enzymatic in vivo biosensor systems have been developed for measuring blood components such as glucose [13–15], cholesterol [16], and lactic acid [17–19]. Energy pathways in birds are dominated by lipid rather than carbohydrate metabolism, and somatic glucose level changes are not as informative as in mammalian system [20]. On the other hand, uric acid is a good indicator of protein catabolism in birds since its presence in the body is a consequence of the breakdown of protein, either from catabolism of body protein or from recently digested proteinaceous food [21]. Birds primarily derive their energy from lipids, but some researchers have shown that there is an increase of the uric acid levels in birds undergoing short flights, which suggests that catabolism of body protein also occurs [22,23]. Elevated uric acid levels after flying events have been observed in flying knots [22] and pigeons [24]. Our group has previously developed a needle-type enzymatic sensor system suitable for real-time amperometric monitoring of interstitial uric acid levels over the expected physiological range in domestic chickens [25]. In that work, besides characterizing the uric sensor performance *in vitro*, the effect of fasting and feeding events on the chickens’ uric acid levels was successfully monitored in vivo with high sensitivity, thereby demonstrating the potential of the sensor system in studying avian systems. However, the significant weight and size of the device limited its use to non-flying chickens whose motions were greatly restricted.

We present here the Lab-on-a-Bird, an implantable lightweight biosensor system suitable for measurement of interstitial uric acid levels in flying birds in vivo. The system consists of a needle-type enzymatic biosensor for the amperometric detection of uric acid, and a two-electrode potentiostat system that drives the biosensor, reads the corresponding output current and wirelessly transfers the data or records to flash memory. In the following sections, we introduce the Lab-on-a-Bird and discuss the preparation of its major components. We then demonstrate the application of the device in measuring uric acid levels in pigeons undergoing flights of varying durations.

## Methods

### System Design and Assembly

**Fig 1:**
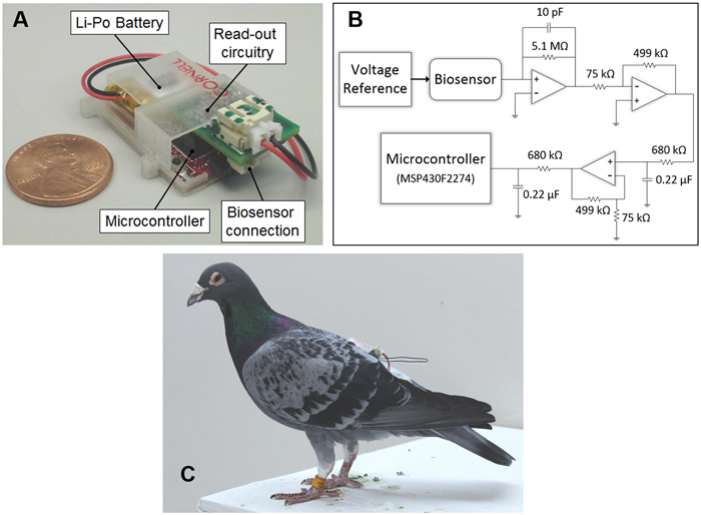
Lab-on-a-Bird system. A) The system consists of microcontroller, read-out circuitry, high capacity lithium polymer battery, and needle-type uric acid biosensor. B) Two-electrode potentiostat system for driving the biosensor and collecting the data. C) A pigeon with Lab-on-a-Bird system installed. The entire system weighs approximately 6.5 g, which is well under 4% of an average pigeon’s weight and allows for long-term tag-attachment without limiting their motion.

The Lab-on-a-Bird system shown in Fig 1a and 1c consists of a needle-type biosensor for the amperometric detection of interstitial uric acid levels, and a two-electrode potentiostat system for driving the biosensor and collecting the data. Those two main components, along with a high capacity lithium polymer battery (3.7V, 40mA; Sparkfun Electronics, CO) are integrated inside a custom package. The entire system weighs approximately 6.5 g, which is well under 4% of an average pigeon’s weight and allows for long-term tag-attachment without limiting their motion. All animal care procedures were approved by the Cornell University IACUC (Protocol No. 2001–0051) with the consultation of veterinarians from Cornell’s Center for Animal Resources and Education and/or by the University of Western Ontario Animal Use Subcommittee (Protocol No. 2010–216).

The lightweight two-electrode potentiostat system shown in Fig 1b combines a low-power microcontroller (“MCU”; MSP430F2274 Texas Instruments, TX), a micro-power analog voltage reference (Intersil, CA), amplifiers (Maxim Integrated, CA), and filter blocks. The circuit drives the biosensor at 0.6 V through the low-power voltage reference and reads the corresponding ultra-low output current of the sensor (1 to 20 nA). A four-layer PCB board was designed and the circuit was built with a minimum number of components to decrease the weight of the total tag by keeping the circuit board area small. The potentiostat system is capable of recording the data to internal flash memory or transmit it to a base station within around 20 meters range using an integrated CC2500 2.4 GHz wireless transceiver. For short-range flight experiments, we transferred the data in real-time to the base microcontroller connected to a remote computer. For mid-range flight experiments, we recorded the data to microcontroller’s flash memory.

### Uric Acid Biosensor Preparation

**Fig 2:**
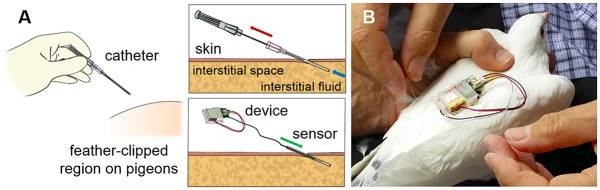
Installation of the Lab-on-a-Bird system. A) The needle-type biosensor component of the Lab-on-a-Bird is inserted into the subcutaneous tissue of the pigeons for interacting with the interstitial fluid. B) A pigeon while installing the Lab-on-a-Bird system on the back of pigeon.

The needle-type biosensor shown in Fig 2a consists of two electrodes of platinum-iridium (Pt/Ir) wire and silver-silver chloride (Ag/AgCl) paste, and a sensing cavity on which uricase enzyme is immobilized. Detailed procedures for the biosensor fabrication and characterization are reported in our previous work [25]. Briefly, a Teflon coated Pt/Ir (10%) wire (0.125 mm o.d.; Advent Research Materials Ltd, Oxford, England) was stripped at one end to create a sensing cavity of 1 mm and the Ag wire (0.1 mm o.d.; Sigma Aldrich, St. Louis, MO) was wrapped around the Teflon coated surface. To create a reference/counter electrode, Ag/AgCl paste (CH Instruments Inc, USA) was applied onto the sensor body. To minimize the effects of interfering chemical species (e.g. ascorbic acid or glucose), the working electrode was coated with an inner membrane composed of Nafion and cellulose acetate. Prior to their in vivo application, the uric acid sensors were characterized in vitro in a 1.5 mL cell that initially contained 0.1M PBS (pH 7.4) with saturated oxygen levels at room temperature (25°C). The solutions of uric acid and/or interferents were injected into the cell and the corresponding sensor output was recorded for the calibration. The sensors were stored in 0.01 M PBS (pH 7.4) at 4°C when not in use.

### *In vivo* Sensor Installation and Calibration

The Lab-on-Bird system was implanted on the back of pigeons as shown in Fig 2b. In order to measure the uric acid levels in vivo, the needle type biosensor component of the Lab-on-a-Bird remained inside the subcutaneous tissue of the pigeons for interacting with the interstitial fluid. For the biosensor installation, a few feathers in the dorsal feather tract, anterior of the uropygial gland, were clipped at their bases and the exposed skin was disinfected using Betadine antiseptic solution (Purdue Pharma, L.P., NJ). As shown in Fig 2a, a 20-gauge catheter consisting of an outer polyurethane layer and an inner puncture needle (Terumo Medical Corporation, NJ) was inserted 1 cm subcutaneously. The inner puncture needle was then removed, and the outer layer of flexible polyurethane was cut just outside the skin surface. This created a cavity into which the needle type biosensor could be inserted. It and the surrounding remnant of the polyurethane catheter were secured to the skin with a ca. 2 cm × 4 cm piece of sterile Tegaderm film (3M Corporate, MN). The custom packaging of the Lab-on-Bird system was attached on top of the sensor on the bird’s back with a Rappole harness [26] made of dental floss or Stretch Magic beading string. The harness ran around the bird’s femurs and stably immobilized the Lab-on-a-Bird on the back of pigeons upon being fastened. Both the biosensor and battery were freely exchangeable, thus one Lab-on-a-Bird circuit could serve in multiple sensing applications with the exchange of fresh sensors and batteries. After installing the tag, we waited for 10 minutes for sensor current to stabilize.

Blood samples from pigeons were taken into heparinized blood collection tubes (Sarstedt CB-300) for biosensor calibration. For mid-range flying homing pigeon experiments, the blood samples were refrigerated immediately upon collection for further analysis in the lab. For short-term flying pigeon experiments, the blood samples were centrifuged at 6000 x g for ten minutes and then plasma was frozen at -86°C. Uric acid concentration was measured using uric acid kit reagent sets (Teco Diagnostics, CA or Wako Diagnostics, VA) and a spectrophotometer (Molecular Devices LLC, CA). Those uric acid levels were correlated to the biosensor output from the *in vivo* experiments using the one-point calibration method [27]. Here, the sensor sensitivity S is the ratio of the sensor output current (I), and the blood uric acid concentration (U). The uric acid concentration was estimated at any time from the sensor current I as U(t) = I(t) / S. The sensor must be calibrated to ensure an accurate relation between sensor output and the corresponding blood uric acid levels. Since sensor sensitivity can change over time [27,28], recalibration of the sensor needs to be performed repeatedly with blood samples. To avoid the recalibration issue at this early stage in free-flight sensor development, we have concentrated on experiments less than two hours in length.

Statistical analyses on changing uric acid levels were made using the *lme4* library in *R* (version 3.0.2). To test the significance of these patterns in the two birds we tested, we conducted two mixed linear model analyses, one for changes during periods of activity and one for changes during periods of rest. The tests consisted of alternating trials of flight and rest, and these trials were characterized by a trial number for each bird. In both analyses, we used uric acid level as the dependent variable and time since the start of the flight or rest trial, trial number, bird ID, and the interaction between bird ID and trial number as fixed effects. We designated trial number, nested within bird ID, as the random effect for these analyses.

## Results and Discussions

To evaluate the performance of the Lab-on-a-Bird system, we implanted the Lab-on-a-Bird on pigeons and used it to monitor changes in uric acid levels under two different flight conditions: short-range and mid-range flying. In order to see the effects of different intensities of exercise on the birds’ uric acid levels and demonstrate the reversibility of the biosensor system, two pigeons were studied under short-range flight and rest cycles in a constrained environment. Here we also examined the wearability of the Lab-on-a-Bird on flying birds. We then tested our device on two homing pigeons for mid-range free flight experiments in the open sky to demonstrate the application of the Lab-on-a-Bird for longer term operations. Lab-on-a-Bird system was removed from animals after the end of the experiments. All animal care procedures were approved by the Cornell University Institutional Animal Care and Use Committee (IACUC) with the consultation of veterinarians from Cornell’s Center for Animal Resources and Education and/or by the University of Western Ontario Animal Use Subcommittee.

### Short-range Flying Pigeon Experiments

**Fig 3:**
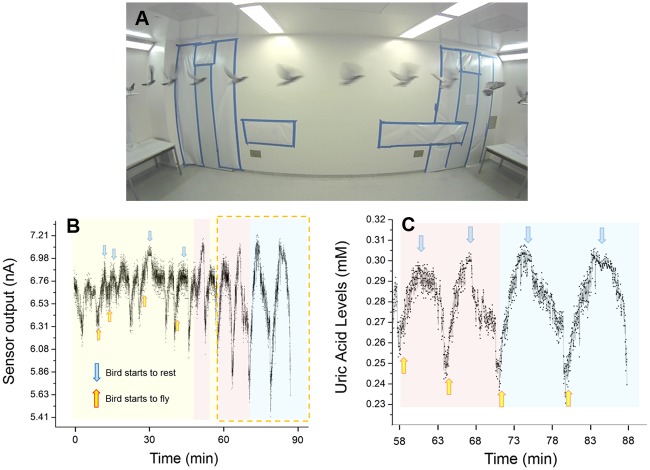
Short-range flight experiments. A) Experimental setup for short-range flight experiments. Bird flies between two platforms at opposite ends of the room. B) Whole experiment data of short-range flight experiments. C) Last part of the experiment calibrated with the blood sample taken at the end of the experiment. Color codes represents as Yellow: 10 flight routines, Red: 20 flight routines, and Blue: 30 flight routines. Uric acid biosensor response increases when bird starts flight routines and decreases when rests.

As shown in Fig 3a, the short-range flight experiments were conducted in a room with two platforms at opposite ends. When released in the room, the pigeons would fly to one of these platforms and stand on them. With the approach of a researcher to that platform, accompanied by a click-note from the researcher, the trained pigeon would fly to the opposite platform on the other end and remain there until the next researcher approach. As a convention, we have designated one flight routine to be the pigeon’s flight to one platform and back to the original platform. We repeated this scheme for 10, 20 and 30 flight routines followed by resting periods. Here the sensor output was related to the uric acid levels by the one-point calibration using blood samples which we took immediately after the experimental flights. As described in more detail in our previous work [25], interstitial uric acid around the sensor is enzymatically oxidized by the immobilized uricase enzyme of our needle-type sensor, producing hydrogen peroxide which is then converted into a measurable current output. The uricase used in this sensor catalyzes the oxidation of uric acid, and uric acid alone, thus we expect that the biosensor output does indeed reflect changes in the pigeon’s interstitial uric acid levels.

There are three kinds of oxidative fuels in working muscles: carbohydrates, lipids, and proteins [29]. For endurance flights, fuel substrates are provided from sources outside the flight muscles, such as adipose tissue [29]. Lipids stored in adipose tissue are the main fuel during long-term flights since it can deliver higher energy [22,24]. On the other hand, for short-term flights, there are some constraints on fat mobilization and delivery, and therefore other fuel types can often be used [29]. Especially at take-off and during short flights, small muscular and hepatic carbohydrate stores are used [22]. Our results indicate, for the first time, that pigeons are also using readily available proteins stored in muscle tissues to fuel short-term flights.

As can be seen from Fig 3b and 3c, the uric acid readings increased when the pigeon started its flight routines and decreased while it rested. Time since the start of the trial had a highly significant effect on uric acid levels for both flying and resting, and this effect was positive during flight and negative during resting trials (slope = 0.03 and p < 0.0001 for flight; slope = -0.03 and p <0.0001 for rest). This regular pattern suggests that the birds are modulating their protein metabolism over very short time scales and in response to short-term changes in behaviour [20,23]. This sort of flexibility is unexpected, but, because this is the first time that data on short-term changes in uric acid levels have been available, there is no body of similar data in birds to which we can compare. We hope that these results will spur much more research in this neglected, and physiologically labile, component of avian biological systems.

It is important to consider whether these results might be explained by an artefact of some kind. We have tried to minimize the effects of pressure changes on sensor response by placing the sensor on the back of the bird and tightly securing the sensor using Tegaderm film. Pressure applied to intradermal glucose sensors can lower the recorded sensor output [30], but this appears to be caused by the reduction of local blood flow. We strongly suspect that blood flow to tissues around the sensor was increased during flight, and that the flow of metabolites to the interstitial fluids would likewise be increased. Our experience with sensors in the lab suggests that stirring around the sensor does not much affect its readings, and any effects of changing flow rates around the sensor will be moderated by the outer polyurethane membrane which serves as a rate-limiting barrier for diffusion [15,18]. On the other hand, increased blood flow to tissues around the sensor during flight could have an effect on increasing sensor output if the device is limited by mass transfer of the uric acid to the interstitial fluid surrounding the sensor. However, stoichiometric considerations reveal this is unlikely. The highest rates of reaction and highest recorded levels of current output (around 7 nA—Fig 3) correspond to a consumption of only 3.6 × 10^-14^ mol uric acid/sec. With an interstitial fluid pocket volume of even 1 μL and normal physiological uric acid concentrations of 0.28 mM (Fig 3), sensor output would take more than 1 hour to consume sufficient uric acid to depress the readings by 50%, which is consistent with what is reported by previous studies [30]. Because the sensor consumes uric acid very slowly, it is not likely to be affected by short-term changes in the flow rate of fluids around it. Rather, even small flow rates are sufficient to surround the sensor with dynamic concentrations of uric acid, and the sharp changes in sensor output we detected thus actually reflect changes in uric acid concentrations per se, not flow rates.

### Mid-range Flying Homing Pigeon Experiments

For the mid-range flying experiments, the Lab-on-a-Bird systems were installed on pigeons that were initially kept 30 miles away from their home and their uric acid level changes were observed while they flew back home. Homing pigeons were trained one week before the actual experiment with dummy tags installed on them, which weighed around the same as the Lab-on-a-Bird devices (~ 6.5 g). This helped them get used to the tag and minimized physiological alterations associated with their flying with foreign systems on the back. Before releasing the pigeons, a blood sample was taken to calibrate uric acid levels from the biosensor. Biosensor read-outs for this longer-distance field trial were recorded to internal flash memory of the microcontroller rather than transmitted over an RF datalink, since these birds would be out of range of our receivers for much of their flights back to the home loft. The data were retrieved from the tags once the birds returned to their home loft.

**Fig 4:**
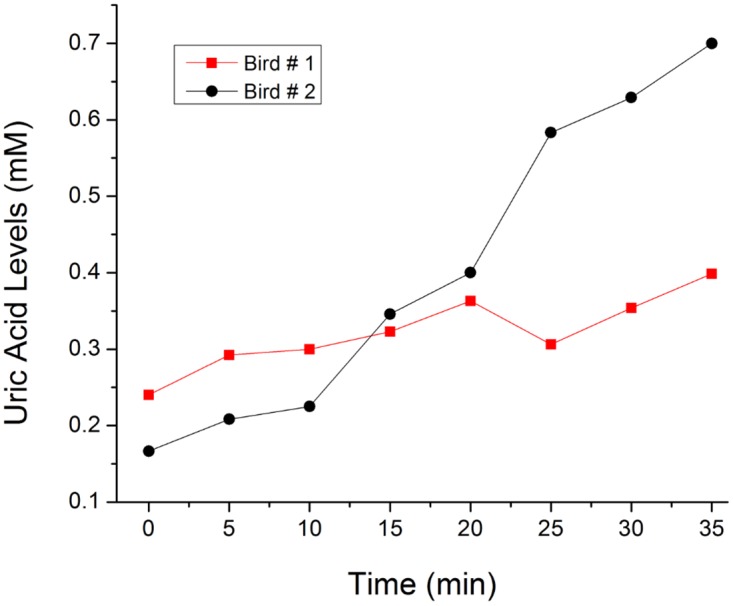
Mid-range flight experiments. The Lab-on-a-Bird systems were installed on homing pigeons and uric acid level changes were observed while they flew back home.

Uric acid is a good indicator of protein catabolism in birds since its presence in the body is a consequence of the breakdown of protein, either from recently ingested proteinaceous food or from catabolism body protein [21]. Although birds primarily derive their energy from lipids, some researchers have shown that there is an increase of the uric acid levels in birds undergoing short flights, which suggests that catabolism of body protein also occurs [22,23]. Elevated uric acid levels after flying events have been observed in flying knots [22] and pigeons [24]. As can be seen in Fig 4, we have successfully observed this uric acid increase in homing pigeons during the first 35 minutes of the pigeon’s flight. Even though there were no technical limitations for longer experimental trials and measurements, the recording time of the tags was cut short when the birds flew into a rain-storm while returning to the loft. The prototype tags used in this experiment were not potted or coated to make them robust to rain, and future versions of the tag could be potted easily to protect them from the elements. However, through the experiment, we have demonstrated that the needle type uric acid biosensor developed here can be used for in vivo measurement of interstitial uric acid levels in flying birds. Here for the first time, a biosensor system was installed on a flying bird to understand the relationship between energy consumption and real-time change in the uric acid levels while they fly.

In the future, the Lab-on-a-Bird system can be further improved by reducing its mass and increasing its environmental robustness, by developing custom circuit boards and water-tight packaging that could reduce the system’s mass substantially. One of the biggest handicaps for deployments requiring uric acid measurements for longer than a few hours is the need to correct for drift in system sensitivity. The single-calibration method used here would need to be augmented by another independent source of information on uric acid levels. Clearly, birds could be recaptured and bled to provide these additional calibration points. The other possible frontier for the Lab-on-a-Bird would be the addition of sensing capability for multiple analytes. Development of multiple-analyte sensing systems would likely lead in a different technological direction, perhaps through the elaboration of multiple-analyte sensing capabilities using microelectromechanical system (MEMS) based technologies [31].

## Conclusions and Future Perspectives

In this work, we have developed the Lab-on-a-Bird, an implantable biosensor system which has the ability to measure uric acid levels in flying birds in vivo. We have successfully observed real-time changes in uric acid levels of flying pigeons. These are the first real-time measurements of a blood chemical, which could be an indicator of their physiological states, in freely flying birds. Our results indicate, that birds modulate their protein metabolism over very short time scales and in response to short-term changes in behaviour. These results confirm the promising application of the biosensor system for real-time in vivo monitoring of birds, which could open a new way of studying birds in flight leading to a better understanding of their ecology and flight biology. In the future, the Lab-on-a-Bird system can be further improved by multiplexing it for the detection of different analytes. Also by decreasing the weight of the system, smaller bird species could be studied.
